# Exploring the Transmission Path, Influencing Factors and Risk of Aerosol Transmission of SARS-CoV-2 at Xi’an Xianyang International Airport

**DOI:** 10.3390/ijerph20010865

**Published:** 2023-01-03

**Authors:** Zhuona Zhang, Xia Li, Keyang Lyu, Xiaoning Zhao, Feng Zhang, Dong Liu, Yonggang Zhao, Fan Gao, Jian Hu, Dongqun Xu

**Affiliations:** 1 China CDC Key Laboratory of Environment and Population Health, National Institute of Environmental Health, Chinese Center for Disease Control and Prevention, Beijing 100021, China; 2 Division of Chemical Analysis, Biology and Medicine, Beijing Institute of Metrology, Beijing 100012, China; 3 Xi’an Center for Disease Control and Prevention, Xi’an 710054, China

**Keywords:** SARS-CoV-2, aerosol transmission, airport, investigation, field simulation experiment

## Abstract

SARS-CoV-2 genetic sequence results collected from native COVID-19 cases who waited or saw relatives off at Xi’an Xianyang International Airport were highly consistent with the imported cases. In order to explore the routes of transmission and influencing factors that may cause the transmission of SARS-CoV-2 at the airport, a field simulation experiment of aerosol diffusion was adopted based on epidemiological survey data and a detailed field investigation of airport structure and ventilation. The results showed that the inbound passengers waited for approximately 3 h in the rest area on the first level of the international arrival area (Zone E). During the period, masks were removed for eating and drinking, resulting in the viral aerosols rising from the first level to the second level with hot air. After deplaning, the inbound passengers handled the relevant procedures and passed through the corridor on the second floor. The local side wall of the corridor adopted fan coil air conditioning, combined with fresh air supply and personnel walking, resulting in airflow flowing to Zone E. After merging with diffused air containing virus aerosol from the first floor, it continued to spread upward to the connected third-layer area. There was a local suspended ceiling on the top of the third floor, but it was approximately 4 m high and connected to the corridor from Terminal 2 to Terminal 3. When the virus aerosol diffused above the Terminal 2–Terminal 3 corridor, where the temperature was low and the air diffused downward, it could cause an infection risk for people passing through the corridor. In addition, the investigation found that the exhaust pipes of the nucleic acid sampling rooms at the international arrival corridor were directly discharged outdoors without treatment. Only one exhaust pipe and poor ventilation in the bathroom in Zone E had a risk of viral aerosol diffusion. Therefore, the international arrival area should be set up alone or separated from the other areas by hard isolation to avoid the existence of communication between different areas that could cause viral aerosols to diffuse with airflow. The toilet ventilation should be increased to avoid the accumulation of viral aerosols at high concentrations. The exhaust pipes of the toilet and the nucleic acid sampling rooms should be equipped with disinfection and efficient filtration devices, and high-altitude emission should be adopted to reduce the risk of virus aerosol diffusion.

## 1. Introduction

With the deepening understanding of SARS-CoV-2, aerosol transmission has been considered another transmission route of SARS-CoV-2 besides droplet transmission and contact transmission [1,2,3,4]. However, the transmission path of aerosol is very complicated. With the continuous variation of SARS-CoV-2 strains, many asymptomatic infection cases have emerged, which makes the virus more prone to occult transmission [5,6]. An international airport is a public place with a large population flow where there are many conditioning and ventilation facilities and the airflow layout is complex, which increases the pressure for epidemic prevention and control in the airport. Xu et al. [7] found that aerosols could spread over a long distance through the supply and return of air through the central air conditioning system in Shenzhen Bao’an Airport. In order to prevent the infection of domestic personnel caused by infected people on international flights, many airports separate the international arrival area from the domestic area by means of isolation, and to prevent the spread of viral aerosol through air conditioning ducts, the Civil Aviation Administration of China revised and issued the eighth edition of “Preventing Spread of Coronavirus Disease 2019 (COVID-19) Guideline for Airports” [8,9] in September 2021. It stipulated that where all air-conditioning systems are used, full fresh air operation mode could be started as appropriate. At present, the return of air through air-conditioning systems in airports is closed, and the full fresh air operation mode is operating.

Although the airport has taken many prevention and control measures to prevent the transmission of viral aerosols, there were still cases associated with inbound passengers at Xi’an Xianyang International Airport. In December 2021, the most serious local COVID-19 epidemic occurred in Xi’an [10], China, since the outbreak in Wuhan, resulting in community transmission triggered by multiple chains of transmission, one of which was related to inbound flights at Xi’an Xianyang International Airport and has attracted widespread attention. It was found by video surveillance of the airport that inbound international flight passengers were at the airport for approximately 3 h to go through relevant procedures, and a total of six COVID-19 cases of Flight PK854 were reported. At the same time, three domestic travelers who stayed at the airport were subsequently diagnosed as positive COVID-19 cases. The virus gene sequencing results for them were consistent with cases on inbound flights. Cases a and b took six minutes to pass through a corridor that was a link between Terminal 2 (T2) and Terminal 3 (T3), and the top of the suspended ceiling was connected with the top of the suspended ceiling of the third floor of the international arrival area (Zone E), and they stayed at the airport for domestic flights for approximately 5 h. Case c was a person who saw a relative off at the check-in counter of T3 for 2 h. There was no actual contact between domestic passengers and inbound passengers, and they appeared at the airport at the same time.

How did domestic personnel at the airport become infected, and what was the virus transmission path? It was necessary to understand the physical structure of the airport, the operation mode of the air conditioning system, the airflow layout and other possible factors to explore the reasons for the transmission of SARS-CoV-2 at the airport. Field measurement and investigation and field respiration simulation experiments to generate virus aerosol were adopted on 23 December 2021, and suggestions on improvement measures against risks that existed in airports were put forward.

## 2. Materials and Methods

**Figure 1:**
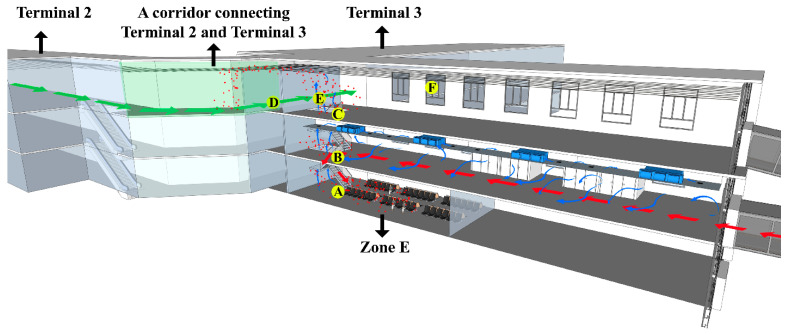
The schematic diagram of architectural composition, airflow layout and walking routes of passengers of the international arrival area of Xi’an Xianyang International Airport and the simulation of viral aerosol diffusion. A: Location of simulating positive cases respiration to generate virus aerosol and monitoring particle concentration changes and environmental factors changes; B–F: Monitoring and sampling points set at the different floors of the international arrival area, the Terminal 2–Terminal 3 corridor and the Terminal 3 hall. Note: 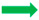 represents walking routes of case a and b who were diagnosed as positive COVID-19 cases. 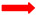 represents walking routes of inbound international flight passengers after getting off the flight. 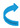 represents direction of airflow. ■ Simulated viral aerosols.

There were three floors in the international arrival area, each of which was approximately 5 m high, and the overall height of the building was 19.2 m. Zone E (first floor) was the hall for inbound passengers’ rest and waiting for their baggage to transfer. The second floor was the international arrival corridor. The third floor was the international departure corridor and the international airport terminal. The three floors were connected by rolling elevators. Figure 1 shows the course of action for inbound passengers and domestic passengers that day. After the arrival of the international flight, inbound passengers need to go through an entry declaration, first temperature measurement, an epidemiology survey, nucleic acid sampling, customs inspection, secondary temperature measurement and border inspection at the international arrival corridor on the second floor, then go downstairs to Zone E (first floor) to wait for the transfer.

**Figure 2:**
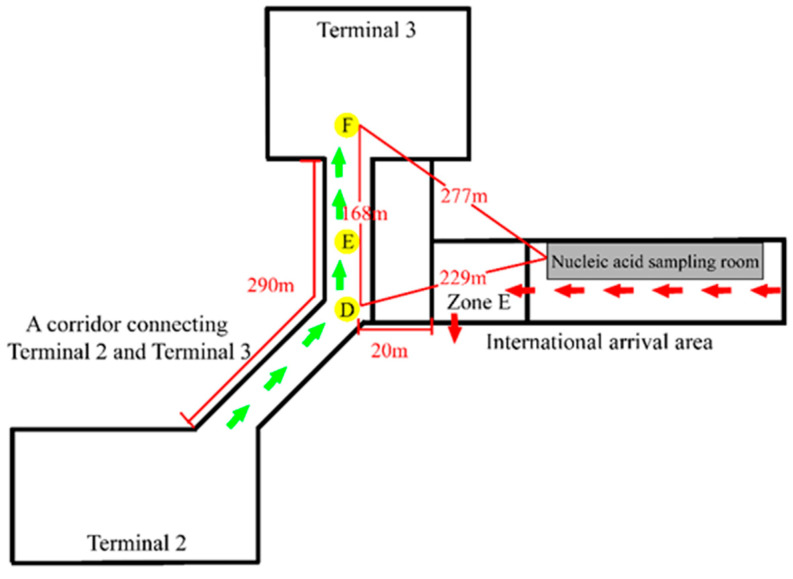
Schematic plan of Xi’an Xianyang International Airport. Distance between different sites is marked in the diagram. Green arrows and red arrows represent walking routes of case a, b and inbound international flight passengers. D–F: Monitoring and sampling points set at the Terminal 2–Terminal 3 corridor and the Terminal 3 hall at different distances.

As the airport staff found, there was always airflow from the exit of the lounge bridge to the staircase at the international arrival area on the second floor. If inbound passengers did not wear a mask standardly, the virus aerosol generated by their respiration would move and diffuse with the airflow. Therefore, people’s walking around and respiration to generate virus aerosol were simulated on the second floor, and monitoring point B was set 12 m away from the occurrence point on the second-floor staircase, and monitoring point C was set on the third floor above the occurrence point. Inbound passengers were concentrated during the period of waiting for transportation to the centralized isolation hotel in the rest area on the first floor (Zone E). They had a behavior of removing their masks for drinking water and eating food. The positive cases could exhale aerosols containing viruses. In order to investigate the infection pathway of local cases, monitoring point D was set at the nearest location to the third floor above the vertical direction of Zone E in the action routes of domestic persons a and b in the T2–T3 corridor; monitoring point E was set 38 m away from point D in the T2–T3 corridor. There were negative-pressure nucleic acid sampling rooms on the second floor of the international arrival corridor. It was stated that the viral aerosol from the positive cases removing their masks for throat swabs discharged from the nucleic acid sampling rooms may spread to the third floor and infect domestic personnel c. Therefore, point F was set at the check-in counter in the T3 hall, where the domestic personnel c stayed (the distance between points D and F was approximately 168 m (Figure 2)).

Fluorescent polystyrene microspheres of different sizes were prepared by the Beijing Institute of Metrology. They did not exist in nature and were consistent with the similar aerodynamic characteristics of the SARS-CoV-2 spike pseudovirus. Therefore, they were selected to simulate particles generated by human respiration [11,12]. The PS microsphere suspension was prepared by simulating body fluid, and the collison aerosol generator (BGI, Inc., Waltham, MA, USA) was used to simulate the respiratory behavior of positive infectors to produce simulated virus aerosols (the volume of exhaled gas was 12 L/min/person, and the number of PS microspheres was 10^11^–10^12^). According to the epidemiological investigation information, the inbound passengers stayed in Zone E for approximately 3 h, so the experimental duration was set to 3 h. During the experiment, the wind speed was monitored to observe the direction and speed of the airflow, PM_2_._5_ and PM_10_ detectors and particle size spectrometries were used for monitoring the concentration changes of particles of different sizes, PM_2_._5_ samplers (100 L/min, ZK-120F, Beijing Zhongke Manatee Technology Co., Ltd., Beijing, China) and biological aerosol samplers (100 L/min, ASE-200p, Shenzhen LemnisCare Medical Technology Co., Ltd., Shenzhen, China) were used for collecting aerosol samples at different sites and the samples were observed by a fluorescence microscope (Leica DM2500, Leica Microsystems Inc., Wetzlar, Germany) to determine the existence of simulants in different regions. Meanwhile, temperature and humidity were monitored. The field direct reading data were statistically analyzed by Origin 9.

## 3. Results

### 3.1. Physical Structure and Ventilation of International Airport

During the epidemic, the airport reconstructed the international arrival area and set up an international arrival corridor on the second floor and Zone E for inbound passengers to go through emigration formalities, and rest and so on, which were separated by an empty, closed area of 15–20 m in length with the T2–T3 corridor. The third floor, where passengers were prohibited from entering, was separated from T3 by hard isolation. After investigation, it was found that the top of the third floor had hollow ceilings, which were approximately 3.6 m from the roof and communicated with the top of the T2–T3 corridor. Airflow was an important factor affecting viral aerosol transmission, and air-conditioning systems played a key role in the airflow layout [13,14]. The airport had a large space, and the air conditioning facilities were different in different regions. T3 adopted a floor radiation and fresh air system that supplied heat with floor radiation and fresh air through the downside air supply. There was no return-air device in the system. The international arrival corridor and Zone E adopted the all-air ventilation system, operated the all-fresh air during epidemics and closed the return air. There were glass curtain walls on both sides of the international arrival corridor on the second floor, where fan coil air conditioning was set.

### 3.2. Risk Investigation on the International Arrival Area

There were 16 nucleic acid sampling rooms set up in the international arrival corridor on the second floor. The nearest distance to point D, where personnel a and b stayed, was approximately 229 m, and the nearest outdoor straight-line distance to point F, where personnel c stayed, was approximately 277 m. There was some risk that the virus aerosols from the infectors removing the masks for throat swabs in the sampling rooms would be discharged through the exhaust pipe directly into the outdoors without any treatment. However, the outdoor distance was too far, though there were open windows on the glass curtain wall of the T3 hall, the possibility of viral aerosol spreading to the T3 hall was small, so the outdoor field simulation experiment of the virus aerosol discharged from the nucleic acid sampling room was not carried out. After inbound passengers arrived, they would go through a health declaration, temperature measurement and so on. During the survey, it was found that the person entered the epidemiological survey area for the special passenger whose temperature was abnormal and then continued to move forward with other passengers for nucleic acid detection and was sent to the first floor through a farther jet bridge and transferred. There was a risk of infecting other passengers. In addition, because the international arrival area only opened a bathroom with only one vent in Zone E for inbound passengers, there was a risk that the aerosol produced by toilet flushing after defecation and urination in positive cases was not discharged in time.

### 3.3. Respiration Simulation Experiments by Fluorescent Microspheres

**Figure 3:**
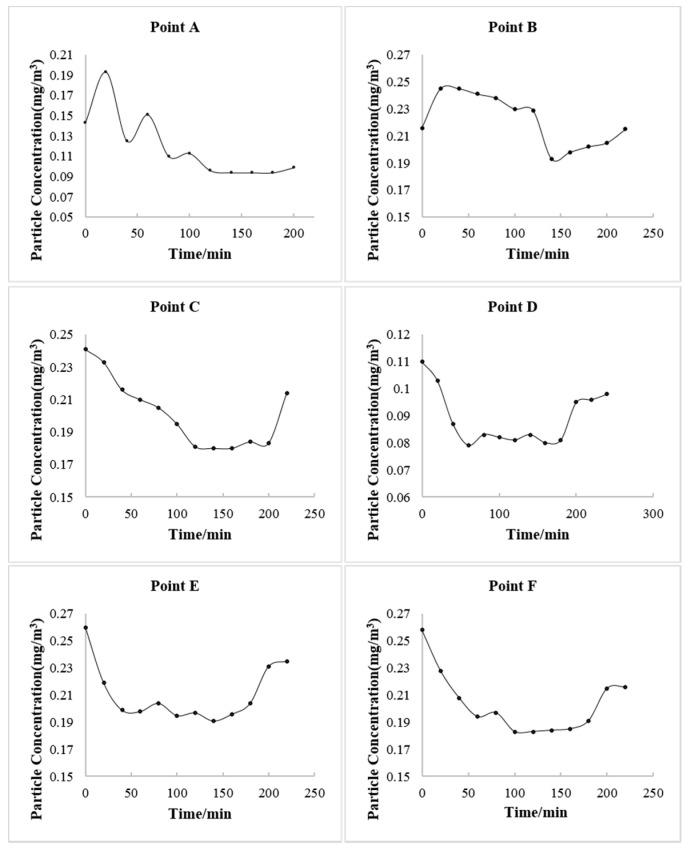
The changes of particle concentration over time at PM_10_ in different points.

**Figure 4:**
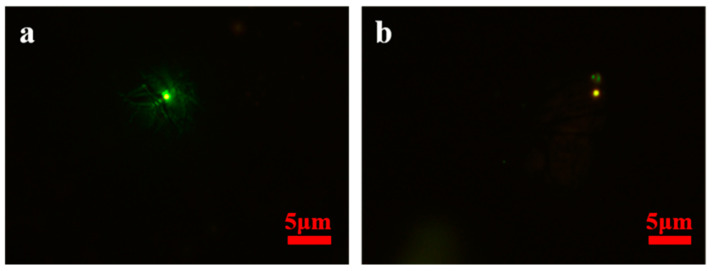
Representative photos of fluorescent microspheres tracked by different sampling methods in different sites. After aerosolization of fluorescent microspheres, fluorescent microspheres (green and yellow) were detected in (**a**) the filter membrane of an air sample using PM_10_ samplers (100 L/min) under fluorescence microscopy and (**b**) the liquid of an air sample using biological aerosol samplers (100 L/min) under fluorescence microscopy.

To explore the transmission path of the virus aerosol, virus aerosols were generated by simulating the respiration of COVID-19 cases in the international arrival area. Figure 3 shows the changes in particle concentration after the occurrence of simulated virus aerosols. The particle number concentration and mass concentration at points A, B, C and D fluctuated with time, and there were uptrends, but they were not obvious (Figure 3, Table S1). During the three hours of the simulation experiment, fluorescent microspheres were observed in the filter membranes and liquid samples at sampling point B in the international corridor on the 2nd floor. In the period of 1.5–3 h, fluorescent microspheres were observed in the filter membrane and liquid samples collected at sampling point C of the third floor and sampling point D of the T2–T3 corridor; no fluorescent microspheres were observed in the filter membrane and liquid samples collected at point E of the T2–T3 corridor and point F at the check-in counter in T3 hall (Table 1, Figure 4). During the experiment, the wind speed at point A was 0.16–0.27 m/s. There had been airflow flowing to the stairs connecting the first and third floors at the international arrival corridor, and the wind speed was approximately 0.35–0.72 m/s, which was consistent with multiple measurements at the airport. The wind speed at point C of the third floor was 0.3–0.46 m/s, and the wind speed at points D and E in the T2–T3 corridor was close to 0.42–0.86 m/s. The wind speed at point F of T3 was 0.79–1.91 m/s. In addition, the ambient temperatures of Zone E, the second and third floors of the international arrival area, were quite different. The temperature of the first floor was significantly higher than that of the third floor, and the temperature difference was close to 7 °C. The temperature difference at point D in the T2–T3 corridor was larger than that in Zone E, reaching a maximum of 11 °C.

## 4. Discussion

### 4.1. Airflow at the International Arrival Area

During the experiment, it was found that the wind speed at the international arrival corridor on the second floor was greater than that of Zone E and the third floor. This corridor adopted fan coil air conditioning for temperature regulation. In open indoor spaces, such as the wholesale market [15], different counters also used fan coil air conditioning. However, because of the open space, the air from the air outlet of a fan coil air conditioner would enter the air inlet of the adjacent air conditioner, resulting in air circulation between the adjacent spaces, and the contaminated air continued to spread far away. The international arrival corridor was also relatively open. The use of fan coil air conditioning made the polluted air continuously flow in the stairway direction, which had been confirmed by the actual measurement results. At the same time, the inbound passengers also moved in this direction and went down to Zone E from the second floor after completing the relevant procedures, which accelerated the flow of the airflow in one direction.

### 4.2. Viral Aerosol Diffusion Caused by Temperature Difference

The airport only opened the first and second floors to provide international arrivals with formalities, rest and so on, and the third floor was vacant and inbound passengers were not allowed to enter. The outdoor temperature in Xi’an was low in December. In order to improve the comfort of passengers, the air conditioning system was opened at the airport. The air conditioning system on the third floor was not open due to no passengers, resulting in a low temperature. Inbound passengers completed the relevant formalities, waited for baggage and transfer in Zone E, and they were more concentrated. Usually, in order to maintain the constant temperature characteristics of the body, the human body will dissipate heat to the environment to achieve thermal balance, which becomes the main heat source of the environment [16,17]. In the winter, the outdoor temperatures are low, and people usually wear heavy clothes when traveling. When entering the room, they would continue to dissipate heat to the room, especially when people gathered and waited, which increased the ambient temperature and generated obvious temperature differences from the first floor to the third floor, causing air to rise from higher temperature regions and diffuse to lower temperature regions. The inbound passengers spent a long time in Zone E for rest. The viral aerosols generated by positive cases through drinking water, eating and breathing after removing masks would accumulate here and spread to the upper space with a temperature difference.

### 4.3. Virus Transmission Caused by Interconnected Headspace at the Airport

The virus aerosol generated by the respiration of inbound passengers while gathering and waiting in Zone E was transmitted upward under the temperature difference, and the use of the fan coil air conditioning and one-way flow of personnel on the international arrival corridor of the second floor promoted the flow of the airflow from the international arrival corridor to the stairway of the same floor and accelerated the diffusion of the virus aerosol. The chimney effect of the vertical penetration of the upper and lower spaces from the first floor to the third floor increased the pull-out effect, which led to the spread of the virus aerosol to the third floor. With the increase in the concentration of aerosols, they would continue to diffuse to the suspended ceiling of the third floor and the connected T2–T3 corridor. There were multiple windows in the corridor, and because the temperature was lower, the upper air contaminated by viral aerosol would diffuse downward. Infection may occur if the mask was not worn or was worn irregularly when the person passed by. Since there was an empty closed area of 15–20 m between the international arrival area and the T2–T3 corridor, the concentration of viral aerosol gradually decreased with the extension of the propagation distance in the transmission process. Therefore, no viral aerosol was detected at the farther point E and the farther point F in the T2–T3 corridor. However, with the extension of the residence time of people in Zone E, the concentration of viral aerosol increased, and there was still the risk of the spread of viral aerosol over a distance due to the airflow change caused by the walking of people in the T2–T3 corridor. The results of field simulation experiments confirmed that the simulated virus aerosol generated by simulated respiration could spread to the second and third floors 12 m away from the occurrence point of Zone E, and spread from the international arrival area to point D of the T2–T3 corridor, but it did not continue to spread to point E of the T2–T3 corridor and farther point F.

### 4.4. Risk Analysis of Nucleic Acid Sampling Rooms

During the SARS epidemic in 2002, it was reported that the virus aerosol spread between buildings in the outdoor environment [18], but its spread occurred under certain conditions. Yu et al. [19] believed in the study of SARS transmission in Amoy Garden of Hong Kong, that the indoor virus aerosol would be discharged into the air shaft outside the building through the exhaust fan and move upward under the buoyancy effect of the warm and humid air in the air shaft. Due to the negative pressure generated by the exhaust fan or the action of wind flows around the buildings, the outdoor virus aerosol would enter the upper apartment units adjacent to the air shaft, which could be transmitted to the B building at 60 m. The airport terminal building environment was not the same. The 16 nucleic acid sampling rooms for inbound passengers were set in the international arrival corridor on the second floor. Field measurement and investigation showed that the distance between the exhaust pipe of the latest nucleic acid sampling room and the glass curtain wall of T3 was approximately 277 m. T3, where domestic personnel c stayed, was located on the east side of the international arrival area, with a glass curtain wall at the top and only eight windows, and adopted no all-air air conditioning system but floor radiant heating and displacement downward air supply [20,21]. The system had no return air, and the maximum wind speed had been set for the air supply in the terminal building, which was not a negative pressure environment. The wind direction on that day was southwest and there was a gentle breeze, and the virus aerosol discharged by the exhaust pipe in the nucleic acid sampling room was easily diluted outdoors. The possibility of inter-building transmission in this long-distance outdoor environment was extremely low. In addition, if the virus aerosol discharged from the second floor was considered to enter the three-floor indoor space above the exhaust pipe again to cause further diffusion, it would need a certain wind direction to achieve this, but the epidemiological investigation did not show the infection of people on the third floor. The experimental results also showed that the virus aerosol generated by indoor simulated respiration did not transmit to point F at 168 m.

### 4.5. Analysis of Other Factors That May Affect Transmission

It has been reported in the literature that the viral aerosol produced by toilet flushing after defecation and urination can also cause the risk of personal exposure and aerosol transmission [22,23,24]. In order to avoid viral aerosol transmission caused by the use of toilets, only a few toilets were open in the international arrival area. In addition, the toilets in T3 of Xi’an Xianyang International Airport and the international arrival Zone E only have an exhaust system without an air supply system, which would not cause the polluted air of the toilet to spread out. Additionally, the toilets in the two areas were not vertically arranged, and contaminated air from the toilets in the international arrival area would not be poured into other vertical toilets via exhaust ducts. There was no walking around of personnel between the two areas, so it was not possible that inbound passengers transmitted the virus to a person who waited and saw others off at the check-in counter at T3 Hall through the use of the toilet or toilet ventilation. However, there was only one vent in the toilet in Zone E, and the virus aerosol produced by toilet flushing after fecal excretion was not easy to be discharged, which had the risk of indoor diffusion. In addition, the strict closed-loop management of crew members and cleaners at Xi’an Xianyang International Airport and the standardized wearing of personal protective equipment made the transmission caused by the lack of standardized wearing of personal protective equipment, such as by cleaners at Nanjing Lukou Airport, impossible [7].

## 5. Airport Risk and Suggestions on Prevention and Control Measures

The airport was an important place for inbound passengers to stay longer after returning. In addition to inbound passengers entering the airport every day, there were also a large number of domestic passengers waiting, sending and leaving the airport, and people were more concentrated. Many airports have established GTC (Ground Transportation Center) as a diversified transportation center that undertakes all kinds of traffic facilities and increases the airport population. Although the airport had taken relevant measures to separate inbound passengers from domestic persons, cross-infection risks still persisted in different regions and personnels.

Airflow layout was the main factor that led to the indoor transmission of virus aerosols. Building layout, the use of an air conditioning ventilation system and personnel movement all affected the indoor airflow layout. Therefore, the following should be practiced in the airport area: (1) The region for inbound passengers to go through formalities and so on should be strictly separated from other regions of the airport and should be set individually or separately by hard isolation means, and the existence of the connection of different regions should be strictly prohibited. (2) The returned air should be closed, and the full fresh air should be used when using a full air conditioning system. (3) The fan coil air conditioning should be avoided in the international arrival corridor to prevent airflow to the connected area. (4) The exhaust pipes set in the indoor nucleic acid sampling rooms need to be equipped with efficient filtration and disinfection devices before discharge and adopt high altitude discharge. (5) It is necessary to increase the exhaust air of the bathroom, install efficient filtration and disinfection devices and discharge at high altitude in areas where inbound passengers of international flights wait to be transferred. (6) After the arrival of international flights, personnel transfer channels should be set up at the corridor bridge nearest to the first temperature measuring point to transfer passengers with abnormal temperatures for the first time as soon as possible.

## 6. Conclusions

In this study, the transmission route and influencing factors of SARS-CoV-2 transmission in airports were determined through a field investigation and simulation experiments. The virus aerosol generated by the respiration of the positive cases in the international arrival area can spread from Zone E (the first floor) with a high temperature to the upper area (the second floor, the third floor and the top of the suspended ceiling) with a low temperature. The use of the fan coil air conditioning and one-way flow of personnel in the international arrival corridor of the second floor promoted airflow to the stairway connecting Zone E. The chimney effect of the vertically connected space made the airflow continue to flow upward, resulting in the spread of the virus aerosol to the upper space of the third floor and the third-floor ceilings, and then continues to spread to the upper ceiling of the T2–T3 corridor with the increase in the concentration of aerosol. The virus aerosol flowed to the area below the T2–T3 corridor, where people passed by because of the lower temperature, resulting in human infection. In addition, there were some risks in the untreated exhaust pipes of the nucleic acid sampling rooms: the toilet with only one exhaust vent in the international arrival Zone E and the delayed transfer of the inbound passengers with abnormal temperatures, but they were not the cause of this transmission.

We used fluorescent polystyrene microspheres consistent with similar aerodynamic characteristics to the SARS-CoV-2 spike pseudovirus to simulate the respiration of infected people to produce viral aerosols, which can verify the transmission pathway and influencing factors of viral aerosols, understand the risk and propose prevention and control measures. Virus infection is a complex process that is related to factors such as virus toxicity, load, activity and the immune status of susceptible individuals and so on. The experiment of the transmission of living viruses cannot be carried out in public places, and the propagation path can be confirmed by using fluorescent microspheres that do not exist in nature. However, fluorescent microspheres are not viruses, and the amount does not indicate the probability of infection.

## Figures and Tables

**Table 1:** Field monitoring and sample detection results at different locations.

Locations	Temperature/ °C	Wind Speed/m/s	Detection Results of Fluorescent Microspheres in Filter Membrane Samples		Detection Results of Fluorescent Microspheres in Liquid Samples	
			0–1.5 h	1.5–3 h	0–1.5 h	1.5–3 h
A	15.9–20.4	0.16–0.27	√	√	√	√
B	14.5–17.6	0.35–0.72	√	√	√	√
C	13.2–14.3	0.30–0.46	×	√	×	√
D	9.4–14.1	0.42–0.86	×	√	×	√
E	9.6–14.3	0.42–0.86	×	×	×	×
F	12.3–15.5	0.79–1.91	×	×	×	×

## Data Availability

Not applicable.

## Supplementary Materials

The following supporting information can be downloaded at: https://www.mdpi.com/article/10.3390/ijerph20010865/s1. Table S1: The changes of particle concentration over time at PM2.5, PM10, 0.3 μm, 0.5 μm, 1 μm, 2.5 μm, 5 μm and 10 μm in different sites.
