# Supplementary material for: Exploring the Transmission Path, Influencing Factors and Risk of Aerosol Transmission of SARS-CoV-2 at Xi’an Xianyang International Airport
Source: Int J Environ Res Public Health. 2023 Jan 3;20(1):865. doi: 10.3390/ijerph20010865 (PMC9820134; doi:10.3390/ijerph20010865)
Supplement: Supplementary file 1 [file ijerph-20-00865-s001.zip › ijerph-2105871-supplementary.pdf]

Supporting Materials

Table S1. The changes of particle concentration over time at $PM_{2.5}$, PM_{10}, 0.3μm, 0.5μm, 1μm, 2.5μm, 5μm, 10μm in different sites .

	Time/min	$PM_{2.5}$/mg/m^3	PM_{10}/mg/m^3	Particle Concentration/particle/L					
				0.3μm	0.5μm	1μm	2.5μm	5μm	10μm
Site A	0	0.081	0.143	130840	45980	12360	1260	100	0
	20	0.111	0.193	132550	43840	10160	970	30	0
	40	0.074	0.125	119840	41280	10510	680	40	0
	60	0.074	0.151	138500	45720	11060	730	30	0
	80	0.067	0.11	119970	39700	9690	760	100	0
	100	0.064	0.113	117630	39490	9370	740	30	0
	120	0.057	0.096	114880	37590	9560	680	40	0
	140	0.054	0.094	111420	36130	8530	560	60	0
	160	0.054	0.094	112530	37500	9440	860	30	0
	180	0.054	0.094	108320	35800	8950	730	30	0
	200	0.059	0.099	123370	39660	9630	850	30	0
Site B	0	0.208	0.216	107000	43950	17250	2650	320	60
	20	0.211	0.245	100100	41400	16440	1890	240	60
	40	0.237	0.245	100500	41740	16650	1890	240	60
	60	0.231	0.241	101460	41560	16270	1510	240	60
	80	0.229	0.238	95050	39100	15400	1680	250	60
	100	0.226	0.23	87900	36480	14560	1740	180	40
	120	0.221	0.229	83890	34770	13870	1420	160	40
	140	0.224	0.193	89120	36300	14080	1500	180	40
	160	0.192	0.198	86850	36730	15080	1640	210	70
	180	0.198	0.202	90550	36610	14040	1600	200	70
	200	0.201	0.205	101420	40570	15270	1860	240	30
	220	0.211	0.215	104140	42560	16590	1700	280	40
Site C	0	0.234	0.241	96130	44560	17730	3260	410	230
	20	0.225	0.233	103450	43720	17650	2630	620	150
	40	0.214	0.216	125060	50420	19210	2500	370	60
	60	0.203	0.21	125710	52270	20400	2900	550	80
	80	0.195	0.205	104920	48620	18730	2950	610	100
	100	0.182	0.195	120830	49580	20210	2720	550	100
	120	0.174	0.181	95530	40920	15660	2480	480	80
	140	0.172	0.18	127020	50800	18420	2400	350	100
	160	0.17	0.18	118350	50220	19300	3010	560	110
	180	0.168	0.184	108300	46210	16810	2730	570	80
	200	0.181	0.183	116100	42860	16500	2670	380	100
	220	0.202	0.214	107730	46200	18260	2530	350	100
Site D	0	0.108	0.11	159330	64910	25150	3620	810	150
	20	0.096	0.103	141450	55000	19700	2960	550	120
	40	0.077	0.087	125150	49680	18460	2800	600	150
	60	0.073	0.079	109310	43910	16650	2670	550	130
	80	0.074	0.083	117360	47080	17810	3100	710	130
	100	0.072	0.082	116420	47200	18160	2850	610	120
	120	0.086	0.081	113720	44870	16510	2630	400	80

	140	0.069	0.083	109700	43700	16330	2370	420	100
	160	0.068	0.08	110100	43550	16080	2280	380	100
	180	0.061	0.081	118960	47250	17670	2620	400	100
	200	0.08	0.095	136820	53810	19670	3020	620	100
	220	0.081	0.096	135350	55810	22030	3380	1050	280
	240	0.084	0.098	129810	57030	24900	3500	720	150
Site E	0	0.246	0.26	175600	73860	30020	5220	470	120
	20	0.209	0.219	130870	54570	21920	3060	330	100
	40	0.185	0.199	124380	53920	22900	2820	420	110
	60	0.187	0.198	131560	55410	22580	3160	430	100
	80	0.193	0.204	140470	57410	22360	3230	420	120
	100	0.186	0.195	120160	51950	21970	2680	370	100
	120	0.187	0.197	123280	51970	21220	3130	400	130
	140	0.183	0.191	108570	46110	19630	2580	380	80
	160	0.184	0.196	117420	48350	19160	2200	300	50
	180	0.192	0.204	130460	56550	24000	3150	460	80
	200	0.22	0.231	140960	59630	24460	3320	480	110
	220	0.222	0.235	160170	67750	27770	3400	480	100
Site F	0	0.258	0.258	150010	65000	30021	4210	870	220
	20	0.214	0.228	123670	54720	23860	3120	460	130
	40	0.191	0.208	117300	51280	22030	2940	400	100
	60	0.19	0.194	113180	49990	21750	3090	430	130
	80	0.191	0.197	113860	49540	21140	2800	490	130
	100	0.187	0.183	107920	47770	20850	3280	540	120
	120	0.181	0.183	106570	50780	24200	2670	430	120
	140	0.177	0.184	105080	47120	20910	2970	490	120
	160	0.178	0.185	104430	45870	19830	2760	420	130
	180	0.183	0.191	109860	47650	20250	2760	400	120
	200	0.208	0.215	121360	53740	23470	3370	490	80
	220	0.21	0.216	126060	55270	23820	3010	430	100
